# Morphological and Ultrastructural Characteristics of Tea Mosquito Bug Antennae, *Helopeltis theivora* Waterhouse (Hemiptera: Miridae) from Hainan, China

**DOI:** 10.3390/insects16070654

**Published:** 2025-06-24

**Authors:** Wenhui Li, Yonglin Liao, Zhufeng Lin, Xuncong Ji, Qi Yao

**Affiliations:** 1 Key Laboratory of Plant Disease and Pest Control of Hainan Province, Institute of Plant Protection, Hainan Academy of Agricultural Sciences, Haikou 571100, China; 15269428738@163.com (W.L.); linzhf123@126.com (Z.L.); 2 Research Center of Quality Safety and Standards for Agricultural Products, Hainan Academy of Agricultural Sciences, Haikou 571100, China; 3 College of Tropical Agriculture and Forestry, Hainan University, Danzhou 571799, China; 4 Institute of Plant Protection, Guangdong Academy of Agricultural Sciences, Guangzhou 510640, China; liaoyonglin2108@163.com

**Keywords:** tea mosquito bug *Helopeltis theivora*, tea plants, antenna, sensillum, scanning electron microscopy, transmission electron microscopy

## Abstract

The tea mosquito bug, *Helopeltis theivora* Waterhouse, (Hemiptera: Miridae) is a significant sap-sucking pest in tropical tea plantations that causes substantial losses in tea production on Hainan Island, China. The morphological and ultrastructural characteristics of *H. theivora* antennae have not been elucidated. Here, we used several microscopy techniques (SDM, SEM, and TEM) to investigate the morphology as well as the setae and sensilla on the antennae of nymphs and adults of *H. theivora*. SDM observations indicated that the antennae of *H. theivora* were filamentous in shape and included four segments: scape, pedicel, flagellum I, and flagellum II. The length of the antenna was approximately twice that of the body and the setae were enriched in flagellum II. The SEM results showed that there were a total of six types of sensilla on the antenna of *H. theivora*, including the sensilla chaetica (SCh), sensilla trichoidea (ST), sensilla basiconica (SB), sensilla coeloconica (SCo), sensilla mammilliformia (SM), and Böhm’s bristles (BB). In particular, there were three subtypes (I, II, and III) of different lengths in SCh and SB, and two subtypes of straight (I) and curved (II) sensilla in ST. The TEM results indicated that diverse internal structures were present in SCh, ST, SB, and SCo, suggesting different functions and different sensory mechanisms of these four main sensilla in the orientation behavior of *H. theivora* on tea plants. These findings provide a theoretical basis for further exploration of the olfactory orientation of *H. theivora* in tropical tea plantations and pave the way for the development of semiochemical-based control options in the future.

## Simple Summary

The tea mosquito bug, *Helopeltis theivora* Waterhouse, is a significant sap-sucking pest of tropical tea plantations in Hainan, China. The morphological and histological characteristics of the nymphs and adults of *H. theivora* antennae were investigated using several microscopy techniques (SDM, SEM, and TEM). SDM observations indicated that the antennae of *H. theivora* include four segments: scape, pedicel, flagellum I, and flagellum II. The length of the antenna was approximately twice that of the body, whereas the setae were enriched with flagellum II. The SEM results showed that there were a total of six types of sensilla on the antenna of *H. theivora*, including the sensilla chaetica (SCh), sensilla trichoidea (ST), sensilla basiconica (SB), sensilla coeloconica (SCo), sensilla mammilliformia (SM), and Böhm’s bristles (BB). In particular, there were three subtypes in SCh and SB and two subtypes in ST. The TEM results indicated that the four main sensilla, SCh, ST, SB, and SCo, might perform different functions and different sensory mechanisms in the orientation behavior of *H. theivora* on tea plants. These findings contribute to further exploration of the olfactory orientation of *H. theivora* in tropical tea plantations and pave the way for the development of semiochemical-based control options in the future.

## 1. Introduction

The tea plant (*Camellia sinensis*) is a perennial monoculture crop that creates an ideal microenvironment to provide continuous food resources for various insect pests [1,2,3]. The tea mosquito bug, *Helopeltis theivora* Waterhouse (Hemiptera: Miridae) is a primary agricultural and forestry pest in the tropics and is widely distributed in China, India, Bangladesh, Kenya, and Australia [4,5,6]. This pest attacks a variety of plant hosts, including tea, cashew, cocoa, black pepper, and cinchona, with tea being one of the most preferred hosts [7,8,9]. Nymphs and adults of *H. theivora* damage the tea plant by piercing and sucking saps from tender shoots, which causes several necrotic black spots on young leaves within a few days, reducing the tea yield and degrading the tea flavor quality [10]. Additionally, oviposition by *H. theivora* can also affect 66% of the harvestable shoots [11]. In the Hainan tea region of China, there are ten generations of *H. theivora* annually without an overwintering period. In particular, the peak of damage by *H. theivora* in tea plantations occurs from August to November, with overlapping generations, which leads to no harvest in extreme cases [12,13].

Insect olfactory receptors are distributed across various parts of the body, including the antennae, head, wings, legs, and reproductive organs [14,15]. Among these, the antennae serve as the primary sensory organs for detecting external odor stimuli [16,17]. The antennae of insects in the family Miridae are typically long and filamentous, and consist of three parts: scape, pedicel, and flagellum. In particular, the flagellum is the longest segment, which can be further divided into two subsegments: flagellum I and flagellum II [18,19]. The morphological characteristics of the antennae in Mirid bugs are usually diverse among different instar nymphs and adults. For example, in *Cyrtorhinus lividipennis*, the antennal length increases during the nymphal stage, with the 5th instar nymphs exhibiting longer antennae than the earlier stages, whereas in adults, females have longer antennae than males [20]. Similarly, the antennal length of *Lygus lucorum* females is significantly longer than that of males [18]. Moreover, in *Adelphocoris suturalis*, annulated protuberances are present on the flagellum and are absent from the scape and pedicel. Meanwhile, the length of antennae in 5th instar nymphs is shorter than that in adults, whereas the antennal length of males is shorter than that of females [21]. In addition, the surface of the insect body is covered with numerous setae that function in chemical perception. For example, the ventral side of the legs of Plecoptera larvae is covered with hairs and setae, which might perform sensory functions [22]. It has also been speculated that pinnate setae might function in cleaning the capture net [23] or serve as receptors for vibration [24].

Different types of sensilla are distributed on the antenna, which plays a key role in the olfactory orientation of insects [25]. The type, quantity, and distribution of sensilla on the antennae in Mirids are highly diverse among the different instar nymphs and adult males and females, as well as among the different segments on the antennae [26,27,28,29]. For instance, a study conducted by Chinta [26] on the antennae of *Lygus lineolaris* indicated that sensilla trichodea (ST), sensilla chaetica (SCh), and the short peg of sensilla basiconica (SB) were exhibited in the last instar nymphs, while the medium-length peg of SB was observed in adults. Furthermore, the total number of sensilla in males was significantly higher than that in females, and the ST and medium-length peg of the SB might possess an olfactory function with existing porous structures and dendrites. In addition, another study on the antennae of adult *Adelphocoris fasciaticollis* showed that there were two subtypes of sensilla for the SCh, ST, and SB [28]. Additionally, two subtypes of ST and one type of SCh and SB were identified on the antennae of *Lygus pratensis* [29].

In the tropics of China, there are seven types of sensilla on the antennae of adult *Campylomma chinensis*, wherein sensilla mammilliformia (SM), sensilla campaniformia (SCa), and sensilla cylindrical (SCy) in males may be involved in the reception of sex pheromones [30]. In adult *Pilophorus typicus*, four types of sensilla, including ST, SCh, Böhm’s bristles (BB), and sensilla squamiformium (SS) were identified, and there were no significant differences between males and females [31]. Additionally, the 16 subtypes of sensilla on the antennae of *Cyrtorhinus lividipennis* were significantly different between nymphs and adults, whereas the multiporous placodea sensilla, SB-II, and sensory pits were only present in adults, and SCh-III was only observed in males [20]. The only previous investigation on the antennae of adult *H. theivora* from cocoa gardens indicated that there were five types of sensilla, namely ST, SB, SCh, SCo, and SM, and the distribution of different types of sensilla varied across each segment on the antennae [19]. We previously described the morphological and biological characteristics of *H. theivora* in tea plantations on Hainan Island, China, and characterized their feeding damage to the Hainan Dayezhong tea cultivar [10,32]. Nevertheless, there are still no reports about sensilla on the antennae of *H. theivora* in tea ecosystems in the tropics of China.

In this study, morphological characteristics of the antennae of nymphs and adults of *H. theivora* from Hainan tea plantations were investigated. Furthermore, we used super-depth microscopy (SDM) to measure setae and applied scanning electron microscopy (SEM) to detect the types of sensilla and their distributions on the antennae of different instar nymphs and adults. Finally, several key sensilla were chosen for further investigation using transmission electron microscopy (TEM) to demonstrate their potential functions. The results of this study support further research on the olfactory orientation mechanisms of tea mosquito bugs in tropical tea plantations.

## 2. Materials and Methods

### 2.1. Insects

Adult *H. theivora* and nymphs were collected from tea plantations located in the Dongchang tea estate, Qiongshan District, Haikou City, Hainan Province (20°00′ N, 110°21′ E), where the large-leaf Hainan Dayezhong tea variety was cultivated. After collection, *H. theivora* were reared on tea shoots in the laboratory in a controlled environment with a temperature of 26 ± 2 °C, relative humidity of 70 ± 3%, and a photoperiod of 12:12 h (light: dark).

### 2.2. Observation of Antennae of H. theivora in the Laboratory

The egg stage of *H. theivora* lasts for 5–13 days [33]. The egg-laden tea shoots were bred individually on water-soaked floral foam and were monitored three times per day in the morning, afternoon, and evening using a stereomicroscope (Nikon SMZ745, Nikon Corporation, Tokyo, Japan). When the egg protruded from the stem epidermis, the eggshell became dark and liquid appeared inside the egg, which signaled the onset of hatching. In that case, the tea shoots were placed under a super-depth microscope (VHX-7000, Keyence Corporation, Osaka, Japan) at X50 magnification to document the entire hatching process. Subsequently, the external morphological characteristics of the antennae of nymphs at the 1st, 2nd, 3rd, 4th, and 5th instars, and adult males and females were recorded using a camera (Canon EOS6D, Canon Inc., Tokyo, Japan).

### 2.3. Determination of Morphological Characteristics of Antennae of H. theivora

#### 2.3.1. Body Length and Antennal Length

VHX-7000 was used to measure the lengths of the body and antenna of the nymphs from the 1st instar to the 5th instar and the adult males and females, respectively. Before taking measurements, the tested *H. theivora* were placed in a transparent tube inside a −20 °C freezer to stun them (approximately 1–2 min for the nymphs and 2–3 min for the adults). Stunned insects were removed from the freezer using tweezers. Under VHX-7000, the body length, length of the total antenna, and lengths of each segment of the antenna (i.e., the scape, pedicel, and flagellum) were measured. Fifteen individuals were investigated for each treatment, and the average values were used for the significance test between the treatments.

#### 2.3.2. Number and Length of Antennal Setae

VHX-7000 was used to measure the number and length of setae on the antennae of the nymphs from the 1st instar to the 5th instar and the adult males and females, respectively. The tested *H. theivora* were first placed in a transparent tube and then stored in a −20 °C freezer. Thereafter, the stunned insects were observed under a VHX-7000 at 200× magnification. The number of setae on the scape, pedicel, and flagellum was calculated for the different instar nymphs and adults, and the lengths of setae inside an area of 10,000 μm^2^ on each segment were averaged. Similarly, 15 individuals were investigated for each treatment, and the mean values were used for the significance test between the treatments. In particular, the four segments on the antenna with the maximum and minimum setae and the longest and shortest setae were photographed.

### 2.4. Scanning Electron Microscopy (SEM)

#### 2.4.1. Sample Preparation

Four types of antennae samples of the early instar nymph, the late instar nymph, and adult males and females were prepared for the SEM test. Under a stereomicroscope (Nikon SMZ745), the intact antennae were excised using a surgical knife and tweezers. These antennae were then immersed in a 70% ethanol solution, and cleaned in an ultrasonic bath for 15 s, which was then fixed in a 2.5% glutaraldehyde solution for 24 h. The fixed antennae were dehydrated using a series of ethanol solutions with increasing concentrations of 75%, 80%, 85%, 90%, 95%, and 100% for 5 min at each concentration. Thereafter, the samples were naturally dried indoors, attached to a conductive adhesive, and sputter-coated with gold in a vacuum using a JYSC-110 sputter coater (Guangzhou Jingying Scientific Instrument Co., Ltd., Guangdong, China), which could be used for SEM observation.

#### 2.4.2. Sample Observation

A Hitachi Desktop SEM (TM4000 Plus II, Hitachi, Ltd., Tokyo, Japan) with MixL, MixH, or BSEM modes was used to observe and photograph the scape, pedicel, flagellum I, and flagellum II on the antennae of early and late instar nymphs and adult males and females, respectively. First, magnifications ranging from 60× to 400× with a scanning voltage of 5 kV or 10 kV were used to observe the segments of the antenna. Subsequently, the MixH mode with a magnification of 1200× to 5000× with a scanning voltage of 5 kV was used to capture the data and analyze the sensilla types. Sensilla types were identified according to Chinta [26] and Schneider [34]. In addition, the lengths of four important sensilla, as well as each sub-type sensillum on the antennae of the early and late instar nymphs and adult males and females, were measured to differentiate maturity among different life stages.

### 2.5. Transmission Electron Microscopy (TEM)

#### 2.5.1. Sample Preparation

Under the Nikon SMZ745 stereomicroscope, healthy and active adult *H. theivora* males and females were randomly selected, and their antennae were removed using a surgical knife and tweezers. The excised antennae were first fixed for 24 h in a Petri dish containing 2.5% glutaraldehyde fixative, which was followed by rinsing three times with 0.1 M phosphate buffer (PB) at pH 7.4, and each rinse lasted 15 min. Thereafter, the samples were fixed in 1% osmium tetroxide prepared with 0.1 M phosphate buffer (PB) at pH 7.4, in the dark at room temperature for 2 h. Again, the samples were rinsed three times with 0.1 M phosphate buffer (PB) at pH 7.4, and each rinse lasted 15 min. After rinsing, the samples were dehydrated using a series of ethanol solutions with increasing concentrations of 30%, 50%, 70%, 80%, 95%, and 100%, for 20 min for each concentration, followed by 2 × 15-min immersions in 100% acetone. The samples were infiltrated with a mixture of acetone and embedding agent 812 at a ratio of 1:1 at 37 °C for 2–4 h, then at a ratio of 1:2 at 37 °C overnight, followed by infiltration with pure embedding agent 812 at 37 °C for 5–8 h. Pure embedding agent 812 was poured into the embedding molds, and the samples were inserted inside and left in an oven at 37 °C overnight.

The overnight-cured molds were placed in an oven at 60 °C for 48 h for polymerization, after which the resin blocks were removed. The resin blocks were then cut into semi-thin 1.5 µm thickness sections using an ultramicrotome (Ultra45) and stained with toluidine blue for localization under a light microscope. After localization, ultrathin sections (60–80 nm) were cut again using an ultramicrotome (Leica UC7, Leica Instruments GmbH, Wetzlar, Germany) and collected on 150-mesh square-hole copper grids. The copper grids were stained with a 2% uranyl acetate-saturated alcohol solution in the dark for 8 min, rinsed three times with 70% alcohol, and then three times with ultrapure water. Subsequently, the copper grids were stained with a 2.6% lead citrate solution in the dark for 8 min, rinsed again three times with ultrapure water, and then slightly dried with filter paper. Finally, the grids were placed in a grid box and dried overnight at room temperature.

#### 2.5.2. Sample Observation

A Hitachi TEM system (HT7800, Hitachi, Ltd., Tokyo, Japan) was used to observe and photograph the cross-sections of the sensilla on the antennae of *H. theivora*. The magnification ranged from 10,000× to 60,000×, with an operating voltage of 80 kV. The cross sections of different types of sensilla were determined according to the sampling position on the antennae, as well as the previous reference [35].

### 2.6. Data Analysis

The experimental data were processed and analyzed using IBM SPSS Statistics 26.0. Significance tests and analysis of variance on the body length and antenna length, number and length of antennal setae, and number and length of sensilla among different instar nymphs and adults of *H. theivora* were performed using one-way ANOVA, followed by Tukey’s post-hoc comparisons.

## 3. Results

### 3.1. Development of H. theivora Antennae

During the observation of *H. theivora* egg hatching, the initially transparent eggs gradually turned orange. Subsequently, the insect’s head emerged from the eggshell. Notably, the antennae originally adhered to the abdomen. As most of the body began to protrude into the tender stem, the antennae started to shake in an attempt to detach from the abdomen. Simultaneously, upon the complete emergence of fresh nymphs, the antennae fully detached and extended forward.

### 3.2. Morphological Characteristics of H. theivora Antennae

**Figure 1:**
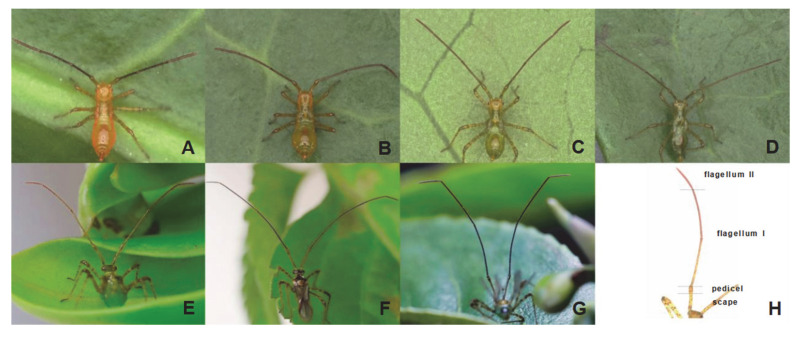
Morphology of antennae of nymphs and adults of *Helopeltis theivora*. (**A**): 1st instar nymph, (**B**): 2nd instar nymph, (**C**): 3rd instar nymph, (**D**): 4th instar nymph, (**E**): 5th instar nymph, (**F**): Adult male, (**G**): Adult female, (**H**): Four segments of the *H. theivora* antenna, including scape, pedicel, flagellum I, and flagellum II.

The antennae of the 1st instar nymphs of *H. theivora* were orange (Figure 1A) and exhibited an orange-red color at the 2nd instar (Figure 1B). Subsequently, the antennal color gradually changed from orange-red to green as it developed from the 3rd instar nymphs to the 4th instar nymphs (Figure 1C,D), whereas it was dark green for the 5th instar nymphs (Figure 1E). In addition, the antennae of *H. theivora* males were black, with the tip of flagellum II being dark red (Figure 1F). Similarly, the antennal color of the females was dark green, whereas the tip of flagellum II was dark red (Figure 1G).

**Figure 2:**
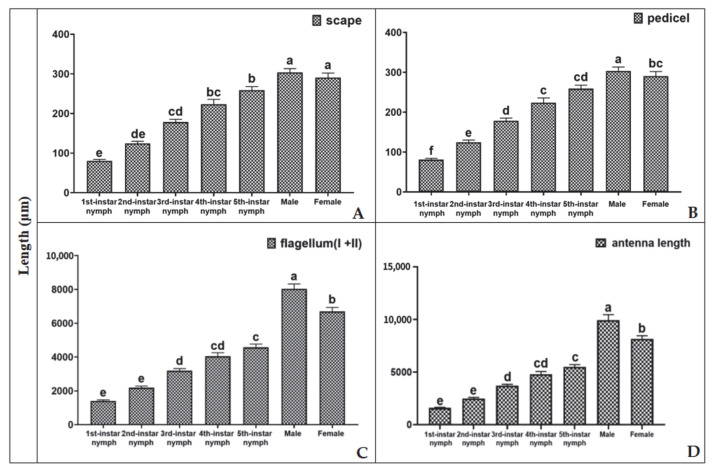
Length of different segments on the antennae of nymphs and adults of *H. theivora*. Data are presented as mean ± SE, and the lengths of each segment: (**A**) scape; (**B**) pedicel; (**C**) flagellum and (**D**) total length on the antennae of *H. theivora* were measured using a super-depth microscope (VHX-7000) at different magnifications. Thirty biological replicates were tested for each treatment. Analysis of variance (ANOVA) was performed using one-way ANOVA, followed by Tukey’s post-hoc test. Different letters above the antennae lengths of nymphs and adults of *H. theivora* indicate a significant difference between treatments at *p* < 0.01.

The antennae of *H. theivora* were filamentous and consisted of four segments: scape, pedicel, flagellum I, and flagellum II (Figure 1H). In particular, the flagellum was much longer than the scape and pedicel (Figure 2A–C). Moreover, the antennal lengths of nymphs *H. theivora* at the 1st instar, the 2nd instar, the 3rd instar, the 4th instar and the 5th instar were 1589.20 ± 57.04 μm, 2488.73 ± 104.00 μm, 3701.59 ± 135.89 μm, 4294.89 ± 256.57 μm, and 5490.49 ± 213.60 μm, respectively, exhibiting a consecutive elongation from the early instars to the late instars (Figure 2D). In addition, the antennae of adult *H. theivora* were generally longer than those of the nymphs. Particularly, the antennal length of adult males was 9932.79 ± 523.20 μm, which was significantly longer than the adult females (8155.01 ± 293.59 μm) (Figure 2D). The overall pattern was the same for the nymphs with the longest antenna observed in males during all stages (Figure 2).

### 3.3. Distribution of Setae on H. theivora Antenna

**Figure 3:**
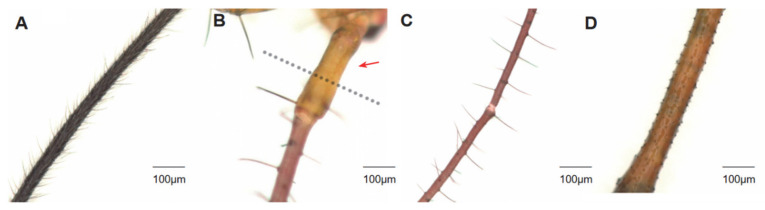
Morphology of setae on antennae of *Helopeltis theivora*. The setae on each segment of the antennae of the nymphs and adults of *H. theivora* were investigated using a super-depth microscope (VHX-7000) at a magnification of 200×. In particular, the maximum setae were presented on flagellum II in adult males (**A**) and the minimum setae on the scape (indicated by the red arrow) in the 1st instar nymph (**B**), while the longest setae were exhibited on flagellum I in the 1st instar nymph (**C**) and the shortest setae on the scape in adult females (**D**).

**Figure 4:**
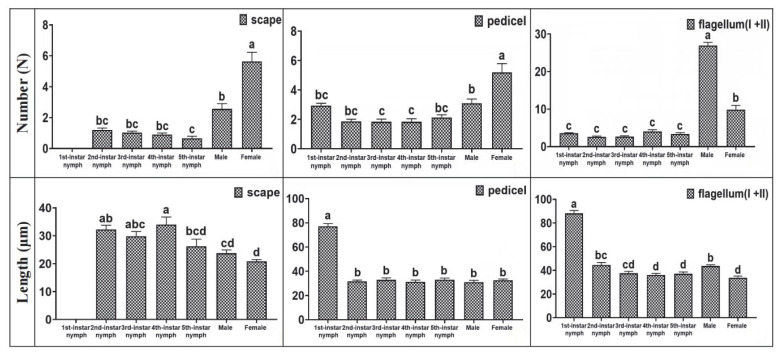
Number and length of setae on antennae of nymphs and adults of *Helopeltis theivora.* Data are presented as mean ±SE, and the number and length of the setae on the scape, pedicel, and flagellum (I + II) of *H. theivora* antennae were measured using the VHX-7000 super-depth microscope (Keyence Corporation, Osaka, Japan) at varying magnifications. Thirty biological replicates were averaged for each treatment group. Analysis of variance (ANOVA) was performed using one-way ANOVA, followed by Tukey’s post hoc test. Different lowercase letters above the number and length of setae indicate statistically significant differences among the treatments (*p* < 0.01).

The setae on the antennae of *H. theivora* were primarily distributed on flagellum II and had a spine-like appearance. There were no setae on the scape of the 1st instar nymphs, whereas the setae developed a reddish-brown color on the pedicel, flagellum I, and flagellum II (Figure 3B and Figure 4). The color of the antennal setae gradually became darker between the 2nd and 4th instar nymphal stages, and then turned dark brown in the 5th instar nymphs and black-brown in the adults. Additionally, the number of setae on the antennae of adults was higher than the nymphs, while the maximum number of setae on the flagellum (Ⅰ + Ⅱ) of males was 26.93 ± 0.85 (Figure 3A and Figure 4). Furthermore, the longest setae presented on the flagellum (Ⅰ + Ⅱ) in the 1st instar nymph with a length of 88.16 ± 2.43 μm (Figure 3C and Figure 4), while the shortest setae were observed on the scape in females with the length of 20.87 ± 0.67 μm (Figure 3D and Figure 4).

### 3.4. Scanning Electron Microscopy on Antennae of H. theivora

#### 3.4.1. Types of Sensilla on Antennae of *H. theivora*

**Figure 5:**
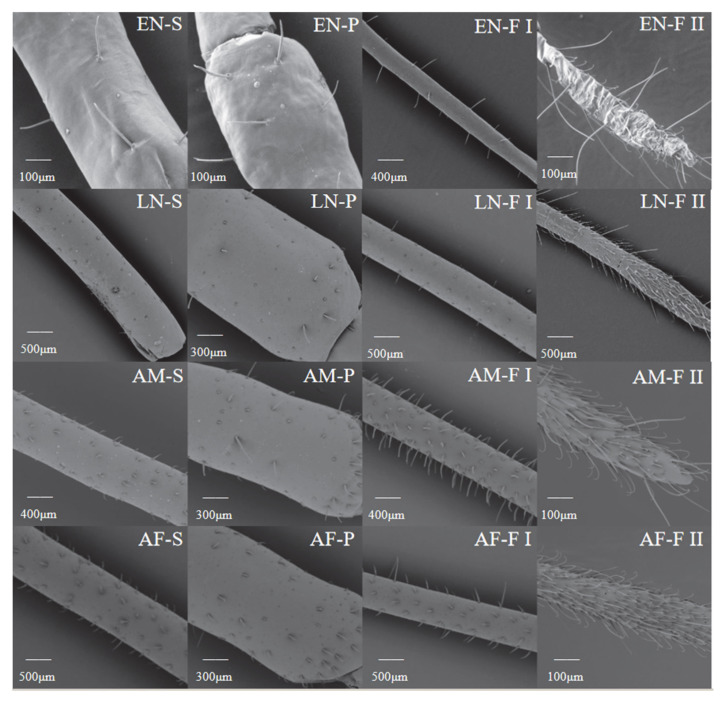
Morphology of each segment on antennae of nymphs and adults of *Helopeltis theivora*. The morphological characteristics of each segment on the antennae of the early instar nymphs (EN), late instar nymphs (LN), adult males (AM), and adult females (AF) were observed using the TM4000 scanning electron microscope (Hitachi, Ltd., Tokyo, Japan) with MixL, MixH, or BSEM imaging modes. EN-S: Early instar nymph scape, EN-P: Early instar nymph pedicel, EN-FI: Early instar nymph flagellum I, EN-FII: Early instar nymph flagellum II, LN-S: Late instar nymph scape, LN-P: Late-instar nymph pedicel, LN-FI: Late-instar nymph flagellum I, LN-FII: Late-instar nymph flagellum II, AM-S: Adult male scape, AM-P: Adult male pedicel, AM-FI: Adult male flagellum I, AM-FII: Adult male flagellum II, AF-S: Adult female scape, AF-P: Adult female pedicel, AF-FI: Adult female flagellum I, AF-FII: Adult female flagellum II.

**Figure 6:**
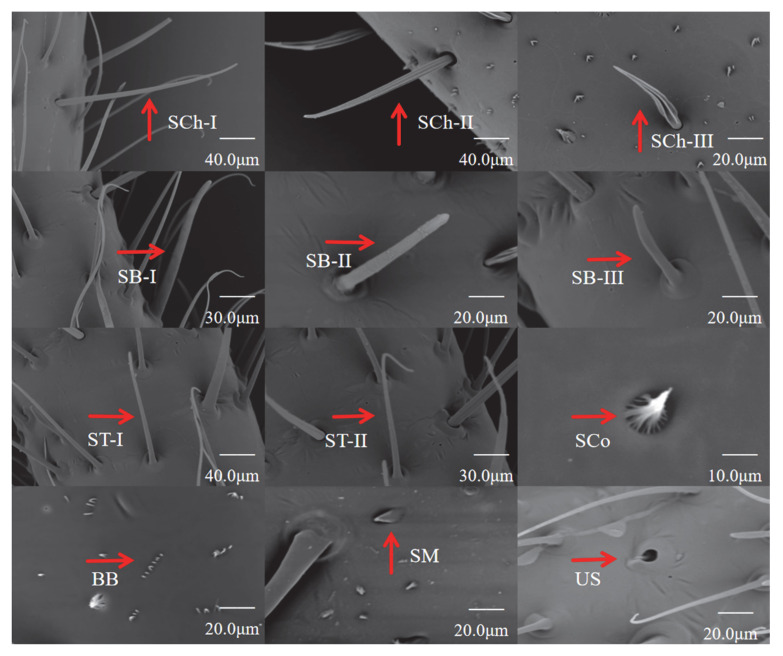
Morphology of different types of sensilla on *Helopeltis theivora* antennae. The morphological characteristics of each type and subtype of sensilla on *H. theivora* antennae were identified using the TM4000 scanning electron microscope (Hitachi, Ltd., Tokyo, Japan) with imaging modes of MixL, MixH, or BSEM. SCh-I: Long sensilla chaetica, SCh-II: Medium sensilla chaetica, SCh-III: Short sensilla chaetica, SB-I: Long sensilla basiconica, SB-II: Medium sensilla basiconic, SB-III: Short sensilla basiconica, ST-I: Long straight sensilla trichoidea, ST-II: Long curved sensilla trichoidea, SCo: Sensilla coeloconica, SM: Sensilla mammilliformia, US: Unknown structure.

There were six types of sensilla on the antennae of *H. theivora* nymphs and adults: sensilla chaetica (SCh), sensilla basiconica (SB), sensilla trichoidea (ST), sensilla coeloconica (SCo), Bohm’s bristles (BB), and sensilla mammilliformia (SM). In particular, there were three subtypes at different lengths in both the SCh and SB, including the long SCh (SCh-I) and SB (SB-I), medium SCh (SCh-II) and SB (SB-II), and short SCh (SCh-III) and SB (SB-III), respectively (Figure 5 and Figure 6). In addition, there were two subtypes of ST, long-curved ST (ST-I) and long-straight ST (ST-II) sensilla (Figure 5 and Figure 6).

#### 3.4.2. Distribution of Sensilla on Antennae of *H. theivora*

Overall, there were significant differences in the type and quantity of sensilla on the *H. theivora* antenna among the different segments of the antenna, among the different life stages, and between the different sexes (Figure 5). First, there was an increasing number of sensilla from scape to flagellum II, suggesting that most sensilla were concentrated on the flagellum. Second, during the nymphal stage, the type, quantity, and density of sensilla continuously increased from early instar nymphs to later-instar nymphs. Third, there was no significant difference in the scape and pedicel between adult males and females; however, there was a denser distribution of sensilla on the flagellum in males than in females.

In the early instar nymphs of *H. theivora*, the sensilla on flagellum II included SCh-III, ST-I, and ST-II, while on flagellum I, the pedicel, and scape all only emerged SCh-III (Figure 6, Table 1). In the late instar nymphs, the sensilla on flagellum II included SCh-III, SB-I, SB-III, ST-I, and ST-II, whereas sensilla on flagellum I presented SCo, SCh-II, SCh-III, and sensilla on the pedicel and scape exhibited SCo, SCh-I, SCh-II, and BB, respectively (Figure 6, Table 1). Moreover, in female *H. theivora*, the sensilla on flagellum II presented SCh-III, SB-I, SB-III, ST-I, and ST-II, while on flagellum I presented SCo, SCh-II, SCh-III, and BB, and both on the pedicel and scape emerged as SCo, SCh-I, SCh-II, and BB, respectively (Figure 6, Table 1). Additionally, in the male of *H. theivora*, the sensilla on the flagellum II exhibited the SCh-SB-I, SB-III, ST-I, and ST-II, while on the flagellum I presented the SCo, SCh-II, SCh-III, SB-II and BB, and both on the pedicel and scape emerged the SCo, SCh-I, SCh-II, BB, and SM, respectively (Figure 6, Table 1).

#### 3.4.3. Length of Sensilla on Antennae of *H. theivora*

There were significant differences in sensilla length between nymphs and adults as well as between adult males and females (Table 2). The lengths of the largest number of antennae, including SCh-II, SCh-III, SB-I, SB-III, ST-I, and ST-II, showed no significant differences between the early and late instar nymphs, which were both significantly shorter than those of the males and females, suggesting more mature sensilla in adults (Table 2). Moreover, there were no significant differences in the lengths of SCh-II, SCh-III, ST-I, ST-II, and SCo between males and females, whereas the lengths of SCh-I, SB-I, and SB-III in males were significantly longer than those in females. Additionally, both SB-II and SM were observed only in males, indicating that the olfactory function of males was stronger than that of females in *H. theivora* (Table 2).

### 3.5. Transmission Electron Microscopy on Sensilla of H. theivora

**Figure 7:**
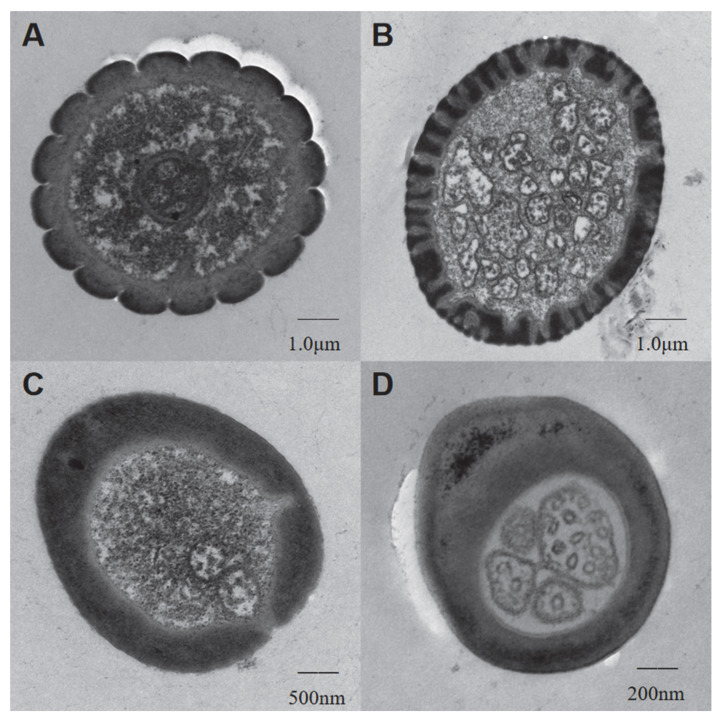
Cross-sections of the four sensilla on the antennae of *Helopeltis theivora,* including sensilla chaetica (**A**), sensilla basiconica (**B**), sensilla trichoidea (**C**), and sensilla coeloconica (**D**), were investigated using the HT7800 transmission electron microscopy (Hitachi, Ltd., Tokyo, Japan) at an operating voltage of 80 kV.

Under TEM, the cross-sections of SCh, SB, ST, and SCo exhibited distinct characteristics on both the cuticular layer and the internal cellular tissue, suggesting the different internal structures of these four main sensilla on the antennae of *H. theivora* (Figure 7). The observations indicated that SCh, SB, ST, and SCo on the antennae might not only play different roles but also perform different sensory mechanisms during the orientation of *H. theivora* in tropical tea plantations.

## 4. Discussion

*Helopeltis theivora* is a notorious insect pest of tropical tea plantations in the Hainan tea region of China. In the present study, we first applied SDM to investigate the morphology of *H. theivora* antennae. Furthermore, we identified the six sensilla (i.e., SCh, SB, ST, SCo, BB, and SM) and some of their subtypes on the antennae, and finally employed TEM to explore the potential orientation function of the four main sensilla (i.e., SCh, SB, ST, and SCo).

The antennae of *H. theivora* are filamentous, structurally including four segments of the scape, pedicel, flagellum I, and flagellum II, which have also been described in other Mirids such as *Lygus lucorum* [18], *Adelphocoris suturalis* [21], *Lygus pratensis* [29], *Campylomma chinensis* [30], and *Nesidiocoris tenuis* [36]. The length of *H. theivora* antennae gradually increased during the nymphal stage and was significantly longer in males than in females. This difference has also been reported between males and females of *H. theivora* from cacao plantations [19], suggesting sexual dimorphism in this pest.

The setae and/or hairs on the antennae can be used to perceive physical contact and air vibrations, and they may also possess thermo-hygroreceptive functions, which are beneficial for detecting environmental changes [24,37]. In addition, antennal setae can also play an auxiliary role in the olfactory localization of insects [22,38]. In this study, we found that denser setae were present on the flagella of both nymphs and adults of *H. theivora*, indicating that the flagellum may play a crucial role in the survival of this pest on tea plants. Furthermore, we also discovered that the number and length of setae on the *H. theivora* antennae were significantly different between the adults and nymphs, as well as between males and females, suggesting different capabilities for physical and chemical perceptions during different life stages. However, whether the antennae setae facilitate olfactory orientation by *H. theivora* on tea plants and how they regulate host localization in tropical tea plantations remain unclear and require further exploration.

The sensilla on the antennae of Mirids was used to perceive and recognize physical and chemical stimulations, which contributed to further localization for landing the host plants, feeding, mating, and/or oviposition [29,39,40,41]. In the present study, six types of sensilla were distributed on the flagellum of the antennae of *H. theivora* collected from tea plantations on Hainan Island, China. This investigation also confirmed that the flagellum played a key role in the olfactory localization of *H. theivora*, which was consistent with another Mirid pest *Campylomma chinensis* [30]. Moreover, the type, quantity, and length of sensilla in adult *H. theivora* were all generally higher than those in nymphs, indicating that sensilla would become more abundant and olfactory capability would strengthen with its growth and development. This has also been observed in another Mirid pest *Adelphocoris suturalis* [21]. Additionally, sensilla distribution was distinct between adult males and females of *H. theivora*. Specifically, SB-II and SM sensilla were only observed in males, suggesting that they might have specific functions such as detecting the female sex pheromone for mating [42].

Different types of sensilla on the antennae of insects typically perform distinct functions such as mechanoreceptors, chemoreceptors, photoreceptors, and phonoreceptors [15,43,44]. According to SEM observations, three types of sensilla (i.e., SCh, ST, and SB) distributed on flagellum II of *H. theivora* antennae, played a decisive role in the perception and recognition of internal and external stimulations for this insect pest. SCh is considered a mechanoreceptor that senses tactile stimulations, including physical contact and environmental vibrations [43,44]. In the present study, three subtypes of SCh were widely distributed on each segment of the antennae in the nymphs and adults of *H. theivora*. In particular, the longest SCh-I was mainly concentrated on flagella I and II, indicating that SCh played the primary role during the tactile sensations for *H. theivora* in tropical tea plantations. This has also been reported in another Mirid pest *Adelphocoris suturalis* [21]. Both ST and SB are considered to have dual functions as mechanoreceptors and chemoreceptors [43,44]. Specifically, the two subtypes of ST and the longest SB-I were mainly distributed on flagellum II of the antennae, suggesting a potential function of the olfactory orientation for *H. theivora* on the tea plant host, which has been described for SB in *Lygus lineolaris* and ST in *Lygus pratensis* [26,29]. However, more efforts are still needed to integrate single sensillum recording (SSR) technology and behavioral assessment to distinguish the functions of these two sensilla for *H. theivora*. In contrast, SCo was mainly distributed in the scape, pedicel, and flagellum I, which might play an auxiliary role in the olfactory orientation of *H. theivora* in tea plantations. Nevertheless, whether there are specific functions for SCh, ST, SB, and SCo, how they independently and/or collaboratively manipulate the host tea-plant finding, and the intraspecific and interspecific communications for *H. theivora* in the Hainan tea region are still awaiting further exploration.

In summary, we applied SDM, SEM, and TEM to systematically investigate the morphological and ultrastructural characteristics of *H. theivora* antennae in tropical tea plantations from Hainan Island, China. However, more research should be conducted on *H. theivora* antennae to address the function of setae, the specific roles of the four main sensilla, and the sensory mechanisms between tea mosquito bugs and tea plants in tropical environments.

## Figures and Tables

**Table 1:** Distribution of different types of sensilla on *H. theivora* antennae.

	Scape	Pedicel	Flagellum I	Flagellum II
Early instar nymph	SCh-I	SCh-I	SCh-I	SCh-I, ST-I, ST-II
Late instar nymph	SCh-III, SCo, BB	SCh-II, SCh-III, SCo, BB	SCh-I, SCh-II, SCo, BB	SCh-I, SB-I, ST-I, ST-II
Adult male	SCh-III, SCo, SM, BB	SCh-I, SCh-II, SCh-III, SCo, SM, BB	SCh-I, SCh-II, SCh-III, SB-II, SCo, BB	SCh-I, SB-I, SB-III, ST-I, ST-II
Adult female	SCh-III, SCo, BB	SCh-II, SCh-III, SCo, BB	SCh-I, SCh-II, SCh-III, SCo, BB	SCh-I, SB-I, SB-III, ST-I, ST-II

SCh: sensilla chaetica, ST: sensilla trichoidea, SB: sensilla basiconica SCo: sensilla coeloconica, SM: sensilla mammilliformia, BB: Böhm’s bristles. In particular, there were three subtypes of sensilla for SCh and SB at different lengths: long SCh (SCh-I), SB (SB-I); medium SCh (SCh-II), SB (SB-II); short SCh (SCh-III), SB (SCh-III). In addition, there were two subtypes of sensilla for ST: long-straight ST (ST-I) and long-curved ST (ST-II).

**Table 2:** Sensilla length on antennae of nymphs and adults of *Helopeltis theivora*.

	Sensilla Length (μm)			
	Early Instar Nymph	Late Instar Nymph	Adult Male	Adult Female
SCh-I	96.38 ± 2.52 ^a^	72.65 ± 2.59 ^c^	82.74 ± 2.38 ^b^	68.42 ± 2.01 ^c^
SCh-II	31.66 ± 1.51 ^b^	31.73 ± 1.49 ^b^	41.23 ± 1.54 ^a^	41.13 ± 1.08 ^a^
SCh-III	10.6 ± 1.06 ^b^	10.60 ± 0.45 ^b^	19.44 ± 0.41 ^a^	20.68 ± 0.39 ^a^
SB-I	39.51 ± 4.74 ^b^	37.89 ± 1.4 9 ^b^	64.92 ± 2.47 ^a^	49.30 ± 1.88 ^b^
SB-II	—	—	37.09 ± 0.64	—
SB-III	13.03 ± 0.73 ^c^	12.53 ± 0.39 ^c^	16.54 ± 0.30 ^a^	15.13 ± 0.21 ^b^
ST-I	33.06 ± 1.68 ^b^	36.14 ± 1.44 ^b^	52.89 ± 1.57 ^a^	52.13 ± 1.24 ^a^
ST-II	29.75 ± 0.83 ^c^	36.31 ± 1.00 ^b^	52.49 ± 1.26 ^a^	50.27 ± 1.00 ^a^

Data are presented as the mean ± SE, and the length of each subtype of sensilla on the antennae of *H. theivora* was measured using scanning electron microscopy (Hitachi Desktop TM4000) at different magnifications. Thirty biological replicates were tested for each treatment. Analysis of variance (ANOVA) was performed using one-way ANOVA, followed by Tukey’s post-hoc test for multiple comparisons. Different lowercase letters in the same row indicate significant differences between the treatments (*p* < 0.01).

## Data Availability

The raw data supporting the conclusions of this article will be made available by the authors on request.
